# Stem Cells in Cartilage Diseases and Repair 2018

**DOI:** 10.1155/2018/3672890

**Published:** 2018-07-24

**Authors:** Jianying Zhang, Shiwu Dong, Wesley N. Sivak, Hui Bin Sun, Peng Chang

**Affiliations:** ^1^University of Pittsburgh School of Medicine, Pittsburgh, PA, USA; ^2^Third Military Medical University, Chongqing, China; ^3^Albert Einstein College of Medicine, New York, NY, USA; ^4^General Hospital of Shenyang Military Command, Shenyang, China

Cartilage is a resilient and smooth elastic tissue that is not hard and rigid as bone but stiffer and less flexible than soft tissues such as muscle, ligament, and tendon. Cartilage is one of the critical tissues found in human and animal bodies including rib cage, ear, nose, bronchial tubes, intervertebral discs, meniscus, elbow, knee, ankle, and the joints between bones [1].

Cartilage plays an important role in the life of human and animals. Healthy cartilage at the joints helps the body move by allowing the bones to glide over each other and protects bones from rubbing against each other. Stress concentration at the joint site is a key issue that can cause cartilage problems such as inflammation, damage, tears, and injuries. Cartilage disorders affect millions of people worldwide. However, the damaged cartilage has little ability for repairing itself due to the lack of blood supply, nerves, and lymphangion [1].

The aim of this special issue is to understand the role of the stem cells in cartilage diseases and regeneration. It has been demonstrated that stem cells play a critical role in tissue regeneration.

For more efficient repair of cartilage, the regenerative medicine provides a variety of trials. Among these, using autologous stem cells to regenerate autologous cartilage is the gold standard in the cartilage tissue engineering. This special issue has focused on the effect of stem cells in cartilage injuries and regenerations. Generally, bone marrow (BM) was the most commonly used source of mesenchymal stem cells (MSCs) [2]. However, low tissue volume and low cell volume have limited the BMSC applications. Searching new stem cell source is a great challenge in tissue engineering and regenerative medicine. Adipose-derived mesenchymal stem cells (ADMSCs) have been used for damaged cartilage regeneration due to adipose tissue that can provide an abundant source of ADMSCs autologously and does not pose the ethical, tumorigenic, or immunogenic risk as presented by pluripotent stem cells. These factors have made adipose tissue a more desirable source of stem cells. S. L. Francis et al. have reviewed several ADSC isolation techniques in this special issue. They developed a rapid one-step isolation protocol that can isolate ADSCs from adipose tissue in 85 min. The authors suggest using this one-step isolation protocol in the context of a surgical procedure.

A novel stem cell population has been isolated from human urine [3]. Human urine-derived stem cells (hUSCs) have several advantages. Firstly, hUSCs show robust proliferation ability and have the capacity for multipotent differentiation [4]. Secondly, hUSCs can be accessed via a simple, noninvasive, and low-cost approach, and thus, surgical procedures are avoided [5]. Importantly, hUSCs isolated from autologous urine have no immune responses or rejection. In this special issue, we would like to introduce an interesting researcher article authored by L. Chen et al. They have demonstrated that human urine-derived stem cells (hUSCs) can be differentiated into chondrocytes *in vitro* and enhanced wounded rabbit knee joint healing *in vivo*.

In this special issue, the potential of synovium-derived stem cells (SMSCs) to regenerate cartilage has been investigated by H. Schmal et al. They tested the effect of SMSCs on chondrogenic differentiation *in vitro* and implanted a SMSC-containing collagen matrix into osteochondral defect of rabbit condyle *in vivo*. SMSC is available in a high quantity, and the isolation procedure does not lead to significant donor site morbidity. The cellular characteristics of SMSC suggest their suitability for cartilage regeneration protocols based on their chondrogenic phenotype including its maintenance after several cell culture passages and their excellent ability to form extracellular matrix [6].

In the current issue, a 3D culture model has been developed for nucleus pulposus (NP) regeneration by Y. Gan et al. They studied the biological effect of a relatively wide magnitude of the dynamic compression (5–20%) on encapsulated MSCs. The underlying mechanotransduction mechanism of transient receptor potential vanilloid 4 (TRPV4) was also investigated.

This special issue also introduces more interesting articles about the effect of environment or stem cell niche on inducing chondrogenic differentiation of stem cells *in vitro* and enhancing cartilage formation *in vivo*. The effect of link protein N-terminal peptide (LPP) as a potential stimulating factor on cartilage stem cells has been studied by R. He et al. The influence of hypoxia-mimetic agent cobalt chloride on chondrogenesis of human MSCs has been investigated by G. Teti et al. in this special issue.

A review article authored by N. K. Dubey et al. provides an excellent summary of the current status of stem cell therapies in osteoarthritis (OA) pathophysiology. The relationships among stem cell types, protein productions, growth factors, cartilage diseases, and cartilage regeneration were outlined and discussed in this review article. In addition, the role of infrapatellar fat pad- (IFP-) derived stem cells in cartilage formation was also described. Furthermore, the effect of exosomes in mediating cellular communication between stem cells and chondrocytes was also summarized in this review article. Finally, the authors indicated the current research limitations of stem cell therapies for cartilage repair including the lack of universal donor cells and the inefficient of reprogrammable approaches to induce stem cell differentiation into cartilage tissue. These limitations will be overcome by genetic modification and gene-editing techniques.

We hope the articles published in this special issue can help researchers comprehend the regulatory mechanism of chondrogenesis and find more useful approaches for enhancing cartilage regeneration and repair.

*Jianying Zhang* *Shiwu Dong* *Wesley N. Sivak* *Hui Bin Sun* *Peng Chang*
